# CircuitBot: Learning to survive with robotic circuit drawing

**DOI:** 10.1371/journal.pone.0265340

**Published:** 2022-03-24

**Authors:** Xianglong Tan, Weijie Lyu, Andre Rosendo

**Affiliations:** 1 Living Machines Laboratory, School of Information Science and Technology, ShanghaiTech University, Shanghai, China; 2 Hamlyn Centre, Imperial College London, London, United Kingdom; 3 Thomas M. Siebel Center for Computer Science, University of Illinois, Urbana-Champaign, Urbana, Illinois, United States of America; Sejong University, KOREA, REPUBLIC OF

## Abstract

Robots with the ability to actively acquire power from surroundings will be greatly beneficial for long-term autonomy and to survive in uncertain environments. In this work, a scenario is presented where a robot has limited energy, and the only way to survive is to access the energy from an unregulated power source. With no wires or resistors available, the robot heuristically learns to maximize the input voltage on its system while avoiding potential obstacles during the connection. CircuitBot is a 6 DOF manipulator capable of drawing circuit patterns with graphene-based conductive ink, and it uses a state-of-the-art continuous/categorical Bayesian Optimization to optimize the placement of conductive shapes and maximize the energy it receives. Our comparative results with traditional Bayesian Optimization and Genetic algorithms show that the robot learns to maximize the voltage within the smallest number of trials, even when we introduce obstacles to ground the circuit and steal energy from the robot. As autonomous robots become more present, in our houses and other planets, our proposed method brings a novel way for machines to keep themselves functional by optimizing their own electric circuits.

## Introduction

Recent developments in the fields of robotic hardware, sensing, and machine learning have led to great progress in robotic applications in industrial settings [1–3]. These advances naturally raise the demand for fully-autonomous, long-lasting and self-evolving robots [4], keeping human efforts further out of the loop. As with living species, robots consume energy to perform different tasks. Industrial robots deployed in the real world are typically powered by an uninterruptible power supply without the danger of running out of energy. For mobile robots, however, have their mobility, operational time and performance limited by the low storage capacity of batteries [5]. This prompts the benefits of an adaptable energy acquisition ability for robots operating in inhospitable environments or away from human aid.

Previous works on self-recharging robots showed a framework for robots to navigate autonomously to a charging station [6]. They use cyclic genetic algorithm to optimize the control program in simulation. In another work [7], researchers built an autonomous robotic system capable of plugging itself into electrical outlets to recharge. Mayton et al. [8] proposed a mobile manipulation platform capable of plugging itself into a standard U.S. electrical outlet. Instead of using vision to assist the process, the plugging was guided entirely by measurements of electrical emissions from the outlets. However, outlets have different standards across regions and they are not always available, especially in the field and unexplored areas. Batteries are more often used in these scenarios, but the connection of cables to batteries by robots is difficult and a fairly unexplored field of research.

Other researchers focus on the design of power systems for self-powered robots. Solar panels are widely used for robots to harvest energy from ambient sources to recharge batteries. They are normally low-cost and light-weight, but suffer from low efficiency and restrictive application environment. In a series of works on energy harvesting [9–11], researchers demonstrate the powering of nanobots from ambient mechanical energy. In a more recent work [12], a novel robotic power system is powered by scavenging energy from external metals. However, in these works the power harvested from the environment is very limited, and it can be hardly used to power any standard robot.

This paper presents a novel approach for robots to leverage ambient electrical power sources and survive in resource-limited, uncertain environments. Instead of using wires or cables, CircuitBot learns to use conductive ink to build its own electrical path to a power supply. The robot learns to avoid environmental obstacles, which tries to steal energy from it, and optimize the new solution. We adopt a novel Bayesian Optimization algorithm, capable of balancing continuous and categorical data sources, and the adopted algorithm outperforms both traditional Bayesian Optimization and Genetic Algorithm given the same number of trials. This paper shows that robots can optimize their own self-drawn electric circuits in very few trials with Bayesian learning techniques, guaranteeing a recharge of their batteries when their survival is at stake.

## Materials and methods

### Conductive ink

Graphene-based conductive ink has shown great potential in printing flexible electronics due to its low cost, high connectivity and versatility, being applicable to textiles [13], papers, and other diverse flexible substrates [14]. Compared to metal-based conductive ink, graphene is low-toxic, environment friendly, easy to make and to store [15]. In this work, we follow the instructions from [16] to fabricate customized graphene-based conductive ink. The ink is made of 5 *wt*% graphene flakes, 0.5 *wt*% graphene dispersion, and 94.5 *wt*% water, which has a sheet resistance of approximately 2 Ω/*sq*.

### Circuit optimization

The conductivity of the connection between the robot and the power source is a major concern. Connection with low resistance would provide lower loss and higher current when powering the robot. The conductivity of the ink can be enhanced by increasing the concentration of the graphene flakes. However, adding more graphene would sacrifice the ink’s viscosity, resulting in the soft pipe being jammed by the ink. Another method is to increase the speed of the ink flow. However, the cardboard on which the robot draws has a very limited water absorption, and with too much liquid on the surface the patterns are unstable and easy to spread; meanwhile, the dry time for the ink to show conductivity increases significantly. The approach we investigate in this work is to create parallel electrical paths, i.e. circular patterns, to reduce the resistance of the connection.

### Experimental setup

**Fig 1:**
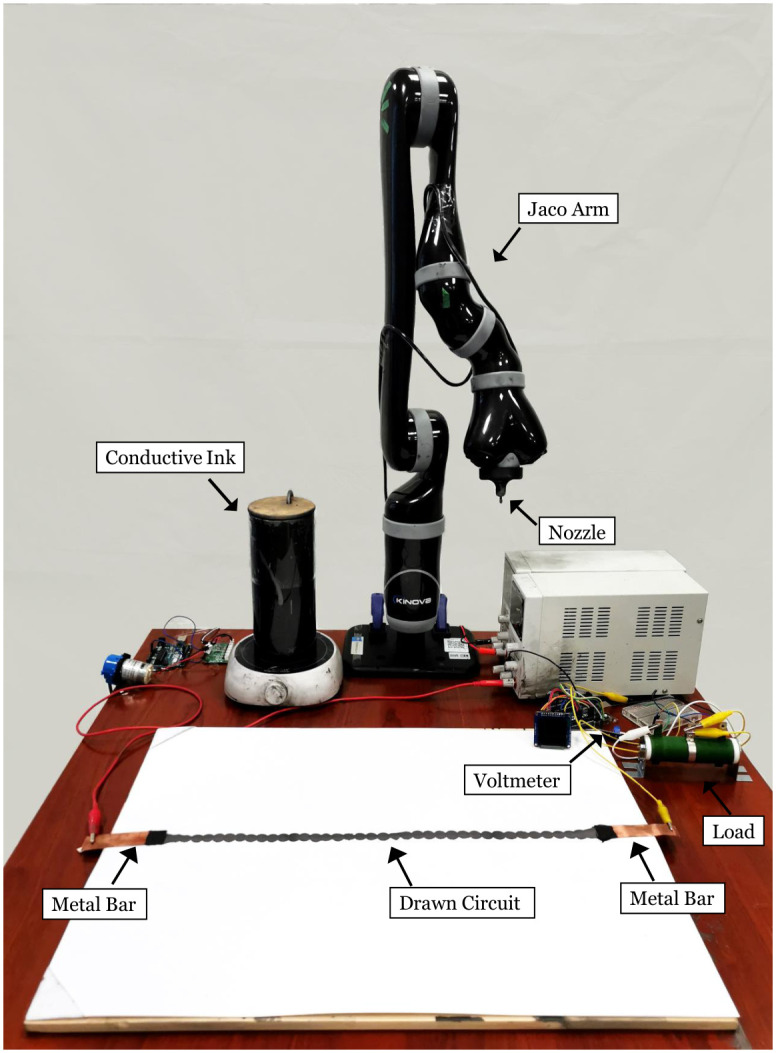
Experimental setup of the circuit drawing robot. The Kinova 6DOF Jaco Arm first moves to an initial Cartesian coordinate with the end-effector (nozzle) at 5 *cm* from the paper. The ROS controller sends instructions to both the arm and an Arduino to begin the circuit drawing. The circuit drawing movements are encoded as a list of Cartesian coordinates and sent to the arm. The Arduino then receives the state of the arm through ROS and sets the on/off of the peristaltic pump to control the ink flow. The robot draws a pattern on the cardboard connecting the two metal bars. The connection starts to show conductivity after the ink dries (30 minutes). A voltmeter is used to measure the load voltage as a feedback to optimize the drawing circuit. The load voltage is shown on an OLED display and the cardboard is replaced after each drawing.

**Fig 2:**
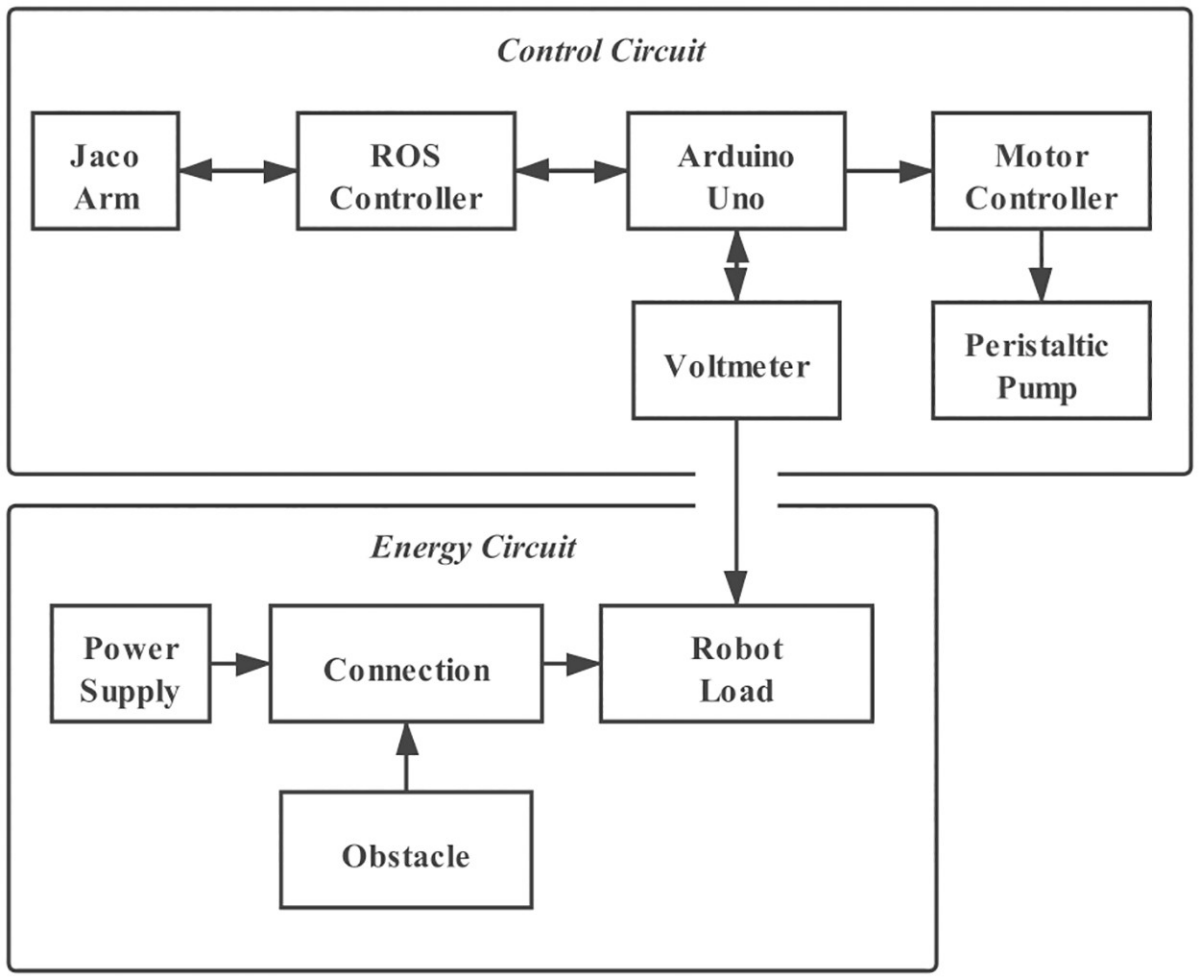
Electrical and control architectures.

The experimental setup is shown in Fig 1. The conductive ink is prepared and stored in a glass jar placed on a magnetic stirrer to prevent the graphene from solidification. The glass jar is linked to a soft pipe which is connected to a peristaltic pump. The pump pushes the ink towards a nozzle which is held by a 3D-printed dispenser at the end-effector of the Kinova 6DOF Jaco Arm. An Arduino Uno is used to control the speed of ink flow. The robot arm is controlled by Moveit (https://github.com/ros-planning/moveit). Two metal bars are fixed on the cardboard, connected to the terminals of the robot and the power source. The circuit block diagram of the robotic system is shown in Fig 2. The voltage of the robotic load (a resistor of 45 ohms) is measured by a voltmeter. The robot draws a circuit which is connected to the load in series, forming a voltage divider.

### Drawing procedure

**Fig 3:**
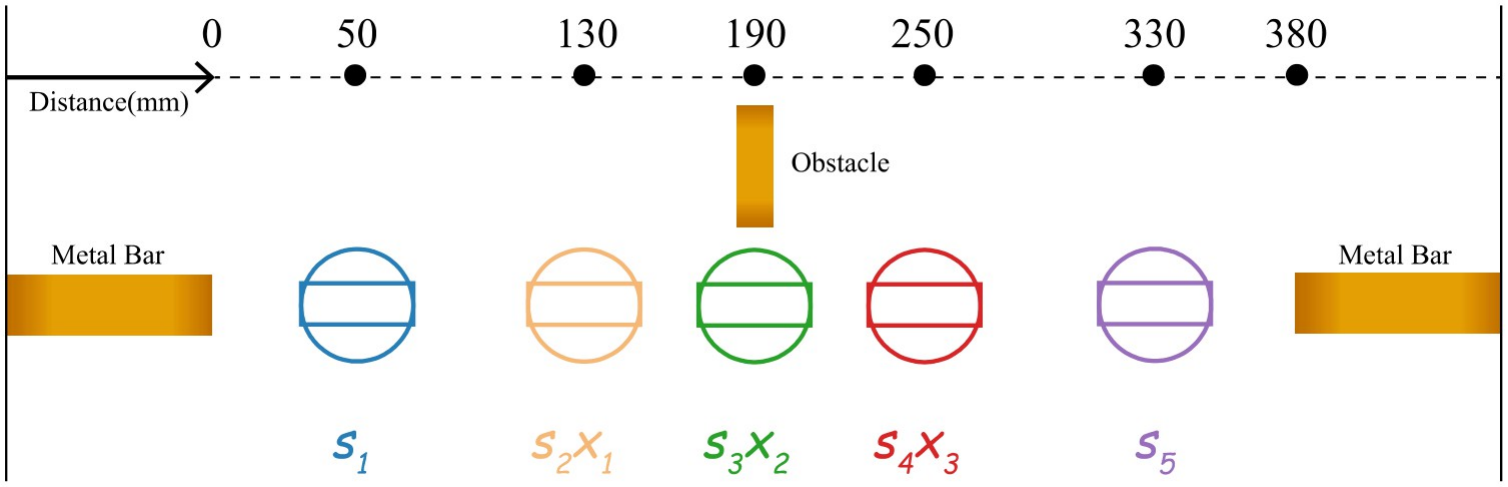
Drawing procedure. Two types of circuit shapes (i.e., line and circle) are selected to form the connection between two metal bars. The circle diameter and line length are both 100 *mm*. In total five shapes *s*_1_ − *s*_5_ are drawn sequentially on a 380 × 100 *mm*^2^ workspace. The central coordinates of *s*_1_ and *s*_5_ are fixed. The central coordinates of *s*_2_ − *s*_4_ are denoted as *x*_1_ − *x*_3_, which have a horizontal displacement range of ±20 *mm*.

The drawing procedure is illustrated in Fig 3, and a list of control parameters was identified, as shown in Table 1. Two metal bars are placed on the cardboard with a distance of 380 *mm*. One is connected to a 30 *V* DC power supply; the other one is connected to a resistive load (45Ω). A total of five shapes are drawn sequentially to connect the two metal bars. The parameter *s*_1_ − *s*_5_ denotes the five shapes drawn from the left metal bar to the right. The parameter *x*_1_ − *x*_3_ are the center coordinates of *s*_2_ − *s*_4_. The voltage of the load is measured by a voltmeter as an indicator of the quality of the connection. We selected two categories of shapes, line and circle, to represent series and parallel circuit patterns. For value of *s*_1_ − *s*_5_, 0 (zero) denotes line and 1 (one) denotes circle. The circle diameter and line length are both set to be 100 *mm*. The workspace has a size of 380 × 100 *mm*^2^, the centers of *s*_1_ and *s*_5_ are fixed. The obstacle is placed in the middle of the workspace. *s*_3_ will touch the obstacle if it is a circle while *s*_2_ or *s*_4_ touches the obstacle if its center is too close to the middle of the workspace. The initial center coordinates of *s*_2_ − *s*_4_ are evenly distributed across the workspace and can displace horizontally for ±20 *mm* to avoid touching the obstacle. All the drawn circuits are dried for 30 *mins* before measurement. The ink flow speed is kept constant and we replace the cardboard after each drawing. We choose the shapes of line and circle to represent series and parallel circuit patterns for certain reasons. The decision between line and circle represents the choice faced by the robot to reach survivability. Among all the shapes we have tried, circle, rectangle, and triangle showed a similar resistance and similar difficulties for the whole circuit to be connected, so we chose circle as a representative. On the other hand, line and curve have a similar resistance and connectivity, and we chose line as a representative. The choice between these two forces the robot to take “gambles” between “safe” and “risky” to optimize its behavior, and this is analogous to how humans and animals make everyday decisions to survive.

**Table 1:** Input variables.

Variable	Name	Quantity	Type	Value
*s*	Shape Indicator	5	Categorical	0, 1
*x*	Central Coordinate	3	Continuous	(-20, 20)

### Bayesian optimization

Bayesian Optimization (BO) has shown great success in optimizing expensive black-box functions [17–22], which is ideal for robotic applications where each experiment is expensive to evaluate. BO is a Gaussian Processes-based optimization approach to find the best configuration of *x* to maximize *f*(*x*)

$$\begin{array}{c} x^{*}=\text{arg}\underset{x}{\text{max}}\;f\left(x\right) \end{array}$$ (1)

BO first builds a surrogate model with Gaussian processes and then uses an acquisition function to choose where to probe for the next observation. The observation then updates the posterior probability distribution (i.e. our current belief of how the system is supposed to behave) and the best set of *x* to maximize *f* will be calculated after iterations.

In this work, however, shape types are encoded as categorical variables while shape locations are continuous, which defeats the common assumption that the BO acquisition function is differential over the input space. Various approaches have been proposed to handle mixed-type (i.e. categorical and continuous) inputs. The simplest method is to use one-hot encoding [23] on the categorical space, which transforms categorical values to continuous ones on which standard BO can perform. However, one-hot encoding significantly increases the dimensions of the search space, making the continuous optimization of the acquisition function much harder [24], which is not feasible for context where there are only limited budget of trials. This work adopts a state-of-the-art BO approach for optimizing mixed-type problems called Continuous and Categorical Bayesian Optimization (CoCaBO) [25].

We consider the problem of optimizing an objective function *f*(*z*) where the input *z* consists of continuous and categorical variables. The aim is to find the best configuration of *z* to maximize *f*(*z*)

$$\begin{array}{c} z^{*}=\left[h^{*},x^{*}\right]=\text{arg}\underset{z}{\text{max}}\;f\left(z\right) \end{array}$$ (2)

where *h* = [*h*_1_, …, *h*_*c*_] are categorical inputs and *x* is a point in a d-dimensional space. The algorithm first builds a multi-armed bandit (MAB) system to select promising categorical values and then applies BO on continuous variables. Authors of CoCaBO chose EXP3 [26] for the MAB algorithm, which is a standard solution for adversarial MAB [27] where the reward distribution is affected by an adversarial agent. For BO, a Gaussian process surrogate model is applied to define the probability distribution of *f*(*x*),

$$\begin{array}{c} f\left(x\right)∼GP(\bar{f}\left(x\right),k\left(x,x^{\prime }\right)) \end{array}$$ (3)

where $\bar{f}\left(x\right)$ is the mean function and *k*(*x*, *x*′) is the covariance function. We have chosen CoCaBO for two reasons: 1) the EXP3 algorithm enables fast selection of promising categories, significantly reducing the number of iterations. 2) CoCaBO shares information across different input types through a special kernel that efficiently leverages all available data. All these features provide advantages for efficient learning which is important for robotic applications where a limited budget of experiments are conducted.

**Fig 4:**
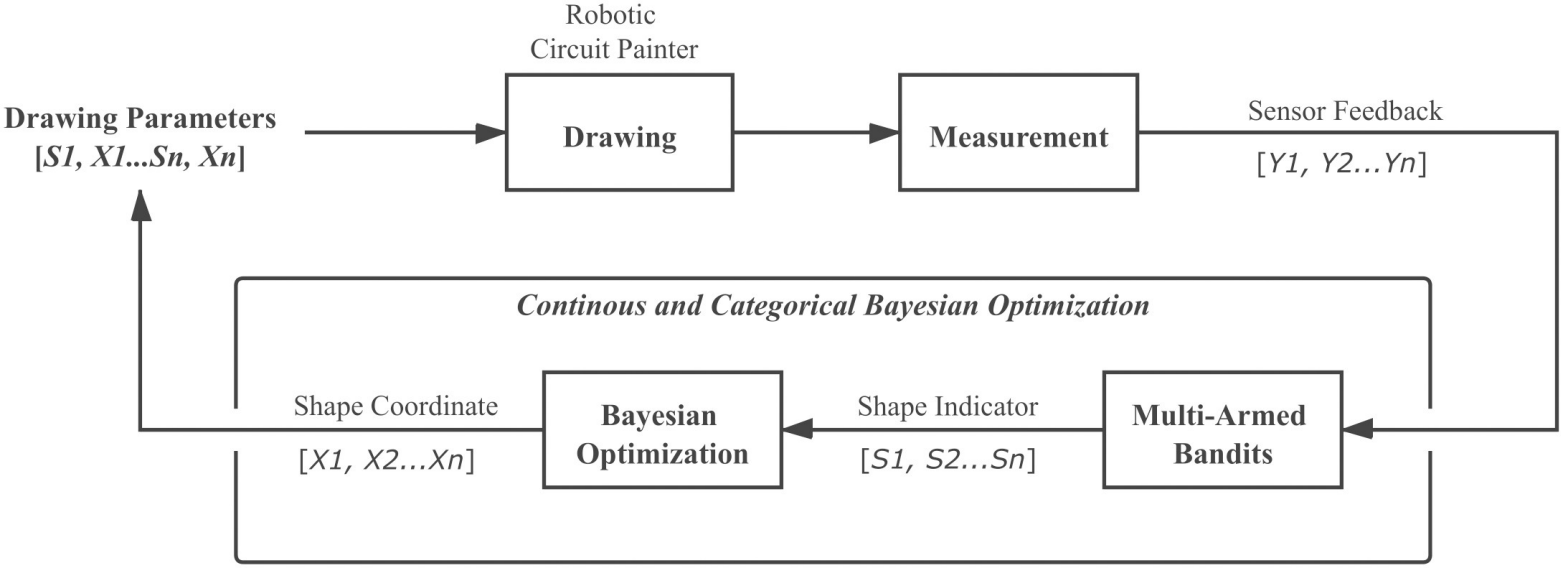
Continuous and categorical Bayesian optimization. The continuous and categorical Bayesian optimization approach we investigate in this paper for circuit optimization.

To evaluate the performance of CoCaBO, we compared CoCaBO with traditional BO. For CoCaBO, we modified the source code in the authors’ GitHub repository (https://github.com/rubinxin/CoCaBO_code) to perform the optimization process. The implementation of the algorithm is shown in Fig 4. For BO, the Gaussian Process (GP) employed a Matern kernel (*nu* = 2.5) and the exploration parameter (*k*) of the acquisition function based on the GP Upper Confidence Bound was set to be 2. In terms of traditional BO, we used a popular BO package (https://github.com/fmfn/BayesianOptimization) and employed the same parameters in CoCaBO. To deal with categorical values, the values suggested by BO are rounded to fall between [0, 1].

### Genetic algorithm

Genetic Algorithm (GA) [28] is a classic and widely-used algorithm for optimization problems. It is inspired by the natural selection process that individuals with high fitness are chosen to be parents to breed the next generation, normally by crossover and mutation. First, a set of variables will form a chromosome and a number of chromosomes will be created as the first generation. Then, each chromosome will be scored by a fitness function, and chromosomes are selected proportionally to the fitness score. Dependent on the crossover rate crossover the values from each chosen chromosome at a randomly chosen point. Based on the mutation rate random the values at a randomly chosen point. Finally, repeating the above steps until reaching the maximum iteration or a threshold. GA naturally copes with both categorical and continuous variables, making it a reasonable reference for CoCaBO. In our experiment, the population size is 5 and the crossover and mutation ratios are set to be 0.5 and 0.1.

### Circuit optimization in uncertain environments

To simulate the uncertain environment in the real world, we create the scenario where an obstacle is placed in the workspace to interfere the energy transmission on the circuit path (shown in Fig 3). The obstacle is a metal bar connected to ground which significantly decreases the voltage that reaches the robot. This obstacle is unseen by the robot, as there are no cameras available.

Our preliminary results have shown that circular patterns are preferred over line connections, as these result in a parallel circuit. However, as circles occupy a larger space, the chance of hitting the obstacle also increases. The task for the robot is to not only avoid touching the obstacle but also draw optimal patterns to receive highest voltage from the power source.

For each of the three algorithms we applied for optimization (CoCaBO, BO, and GA), a total of 30 circuit patterns were drawn. The random seed is set the same for all three algorithms. The first five patterns of BO and CoCaBO are manually set, which are identical with the five individuals in the first generation for GA. These five patterns were selected that the circuit would touch the obstacle leading to a low load voltage instead of patterns with high voltage in order to better observe how optimization processes.

## Results

### Linear versus circular connections

We first conducted an investigation to examine the reliability of line and circle circuits. We conducted 20 trials with each shape type centered at different locations across the workspace. The results obtained are shown in Table 2. The length and resistance of lines and circles showed a high repeatability, indicating a strong reliability on the pump-nozzle ensemble during the drawing procedure.

**Table 2:** Drawing reliability.

Shape Type	Resistance (Ω)	Length (*cm*)
Line	20±0.7	10±0.2
Circle	16±1.2	10±0.6

**Fig 5:**
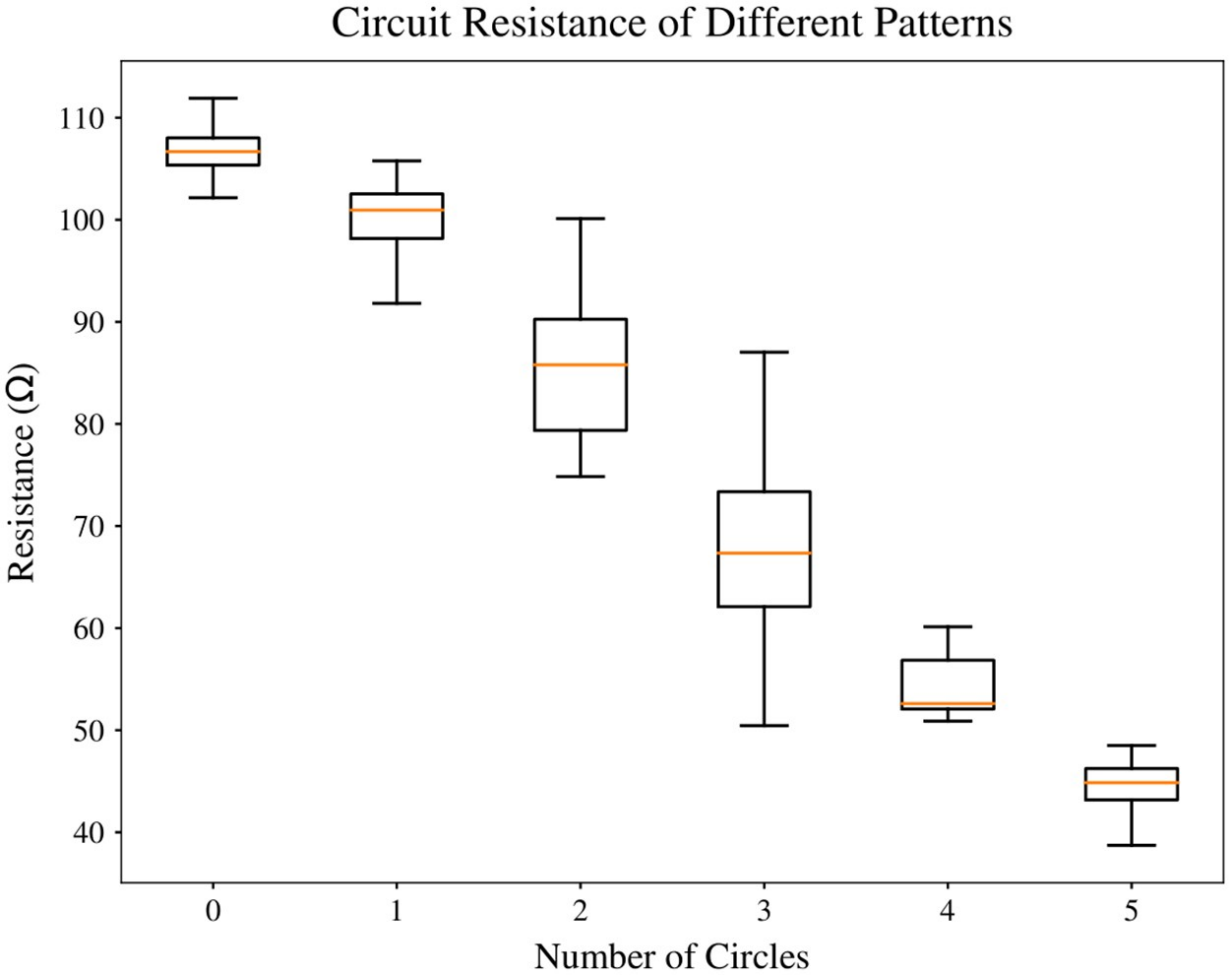
Circle number versus resistance. The overall resistance of connections decreases as there are more circles in the patterns. Significant reduction in resistance happens with 3 or more circles as more bridge circuits are created when circles intersect with each other.

We then tested the resistance of different patterns without the obstacles. The relationship between resistance of circuits and circle numbers is shown in Fig 5. We can see that moderate improvements are achieved by having one or two circles in the circuit patterns as circles have smaller resistance than lines (parallel circuit). Interestingly, we observe significant improvements in the mean resistance when the circle number increases from two to five. This can be explained by the formation of bridge circuits as the result of circle-circle intersection. Note that patterns with three circles present high variance in the resistance because the number of bridge circuits can change from zero to two, depending on the order of circles. Having more circles is more likely to create bridge circuits, which significantly reduces the overall resistance of the connection. This phenomenon is not observed when circles are tangent to each other, as the circular path tends to be longer than the path created by a straight/semi-straight line. When compared to the original single line connection the connection with circles presents half of its resistance.

### Circuit optimization in uncertain environments

We add obstacles to our experiment to challenge the robot with a competitive and uncertain environment. The obstacle is connected to the ground, and once the circuit connects to this obstacle the voltage decreases significantly. As we have known from the results above, the load voltage is mostly affected by the number of circles. The optimal patterns in this setting are likely to have as many circles as possible while avoiding touching the obstacle. In order to achieve optimal load voltage, the best strategy is to draw a line for *s*_3_ and draw circles for *s*_2_ and *s*_4_ while keeping *x*_1_ and *x*_3_ far away from the obstacle. This strategy requires shared information between categorical and continuous domains.

**Fig 6:**
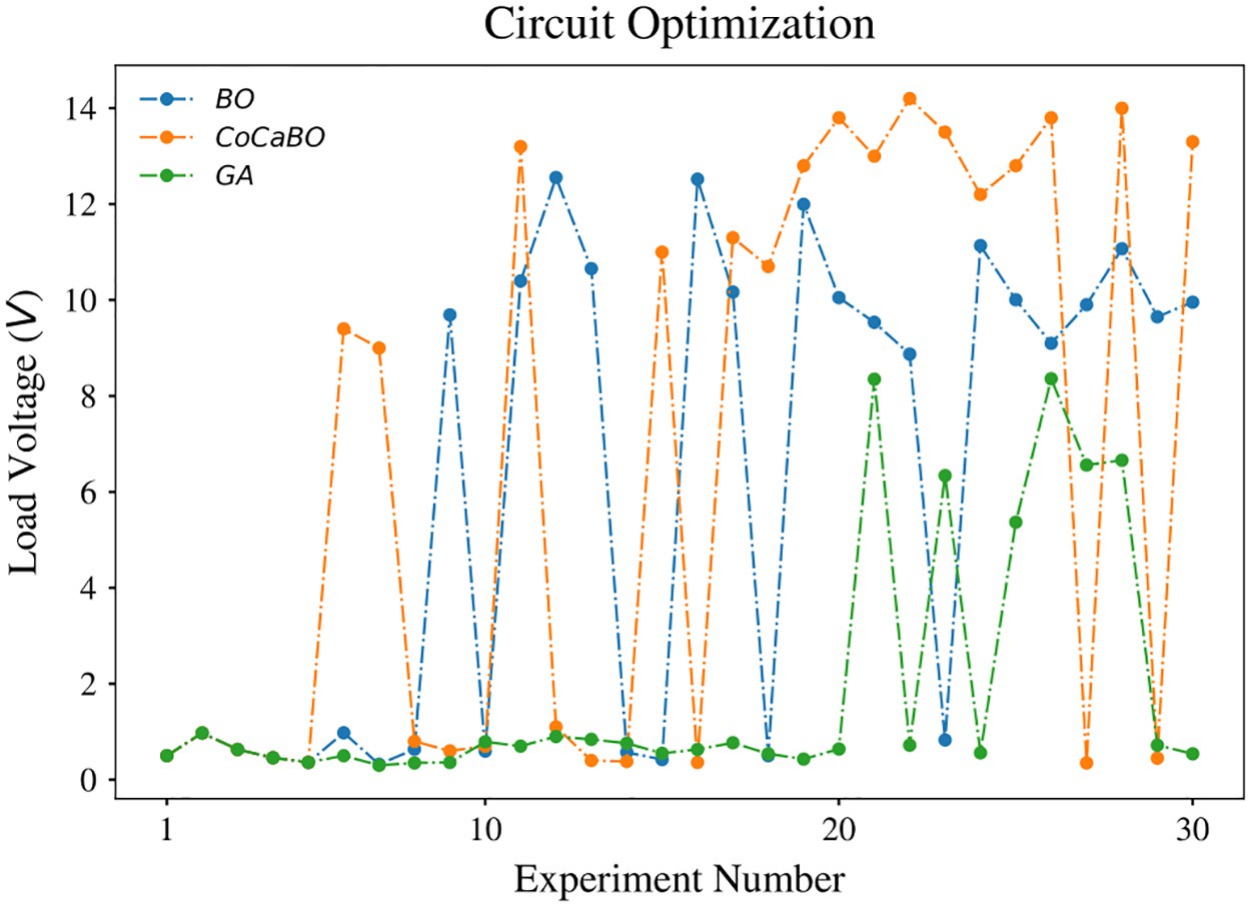
Result of circuit optimization with one obstacle. Patterns that make contact with the obstacle result in a low load voltage. Starting from low voltage values, CoCaBO achieves high load voltage values and outperforms both BO and GA (12.8% and 71.9% higher, respectively). While simultaneously coping with discrete and continuous choices GA shows the worst performance in this experiment.

**Fig 7:**
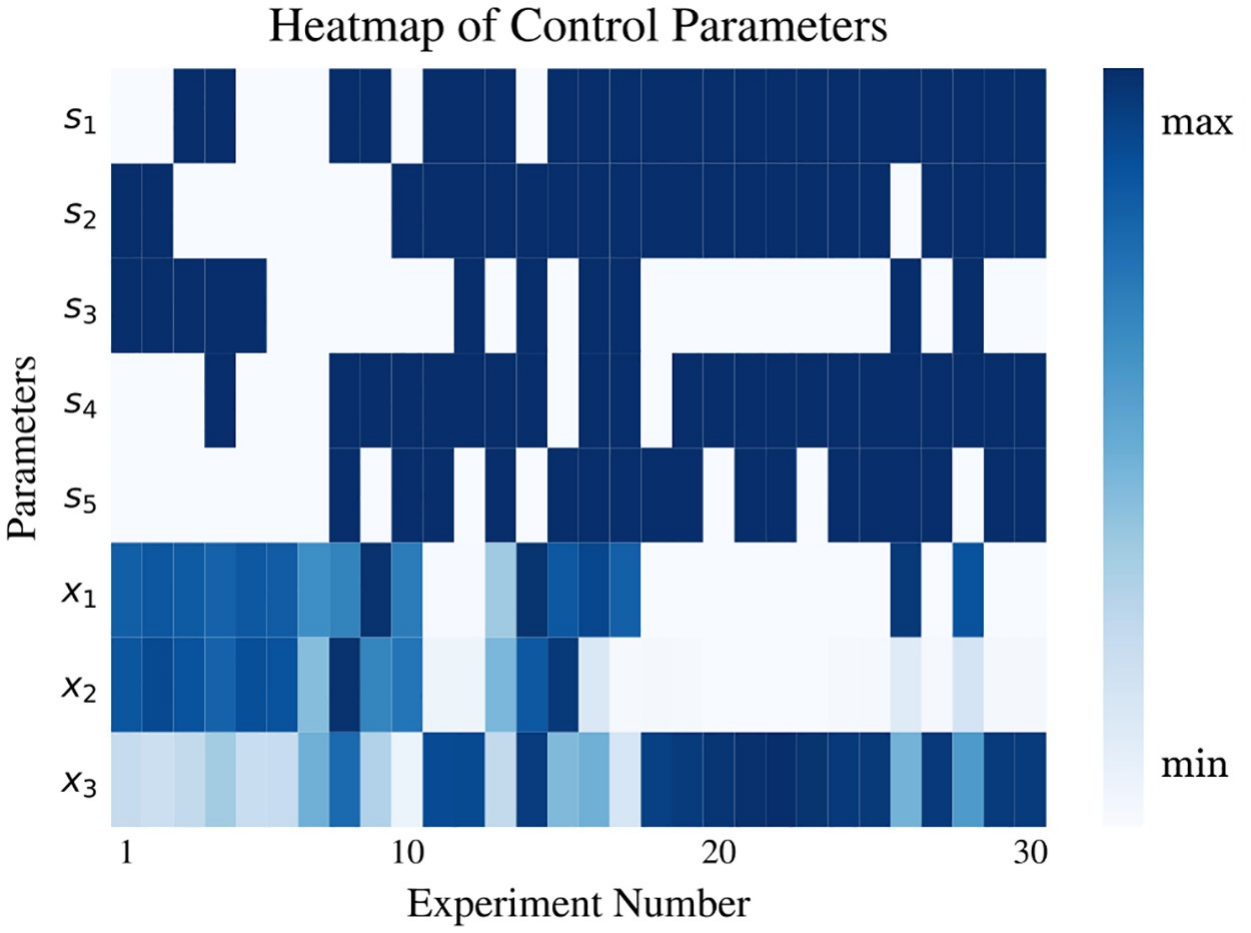
Heatmap of control parameters from CoCaBO. For *s*_1_ − *s*_5_, dark blue denotes circles and white denotes lines. The obstacle can be avoided if *s*_1_, *s*_2_ and *s*_3_ are all lines or *s*_1_ and *s*_3_ are circles centered away from the middle of the workspace. When *s*_2_ and *s*_4_ are both circles, the robot learns to constrain *x*_1_ and *x*_3_ in a small search area to prevent connecting the obstacle.

The observed load voltage is shown in Fig 6. It is clear in the figure that CoCaBO (orange line) outperforms BO and GA not only in finding the highest voltage but also in the earlier increase in output. The voltage of the optimal pattern CoCaBO found is respectively 12.8% higher than BO and 71.9% higher than GA. A significant increase in the load voltage is seen before iteration 15 for CoCaBO and BO, while GA only reaches a relatively high value after performing 20 drawings. To have a better view of the parameters chosen by CoCaBO, a heatmap was plotted (Fig 7).

**Fig 8:**
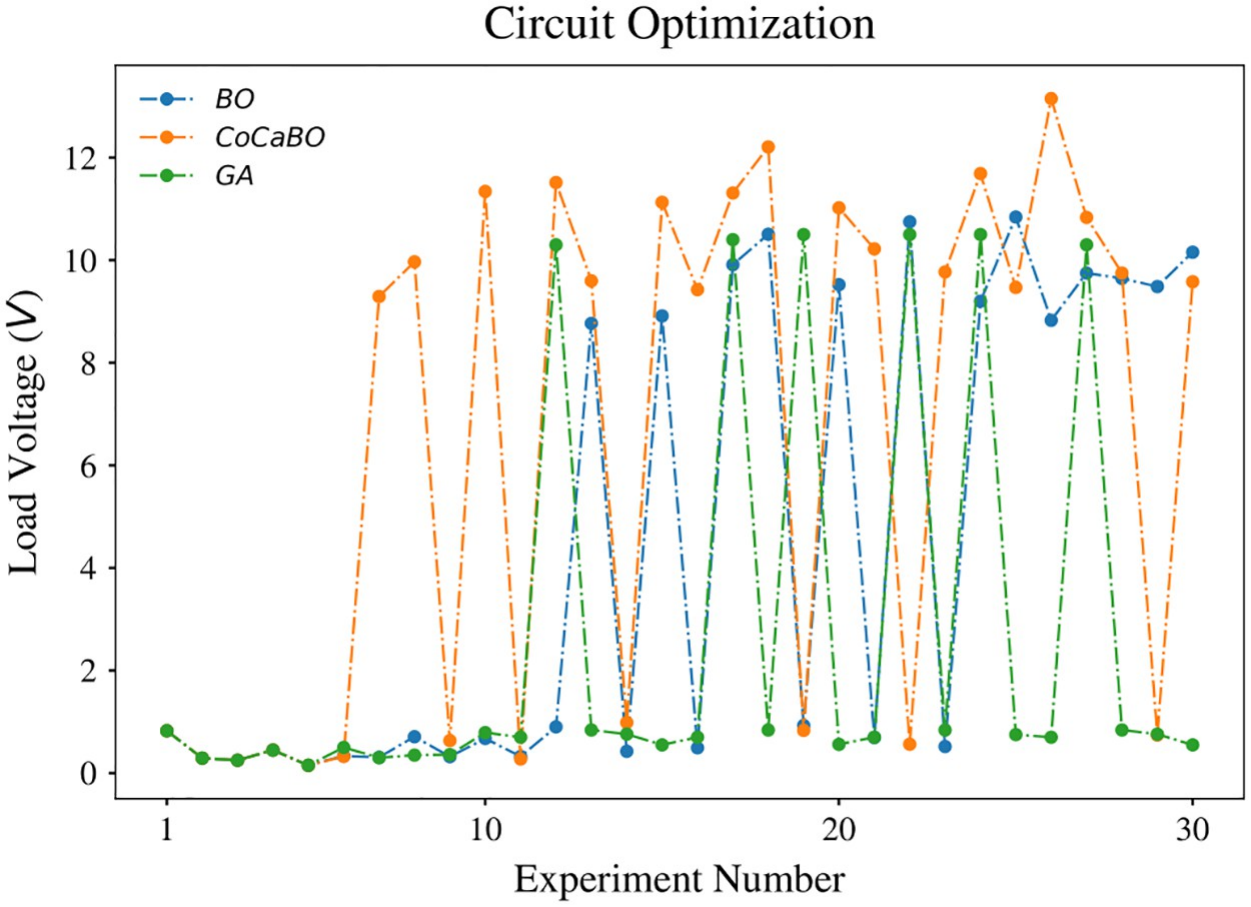
Result of circuit optimization with two obstacles. Similar performance is achieved by CoCaBO that outperforms both BO and GA (21.3% and 25.7% higher respectively).

**Fig 9:**
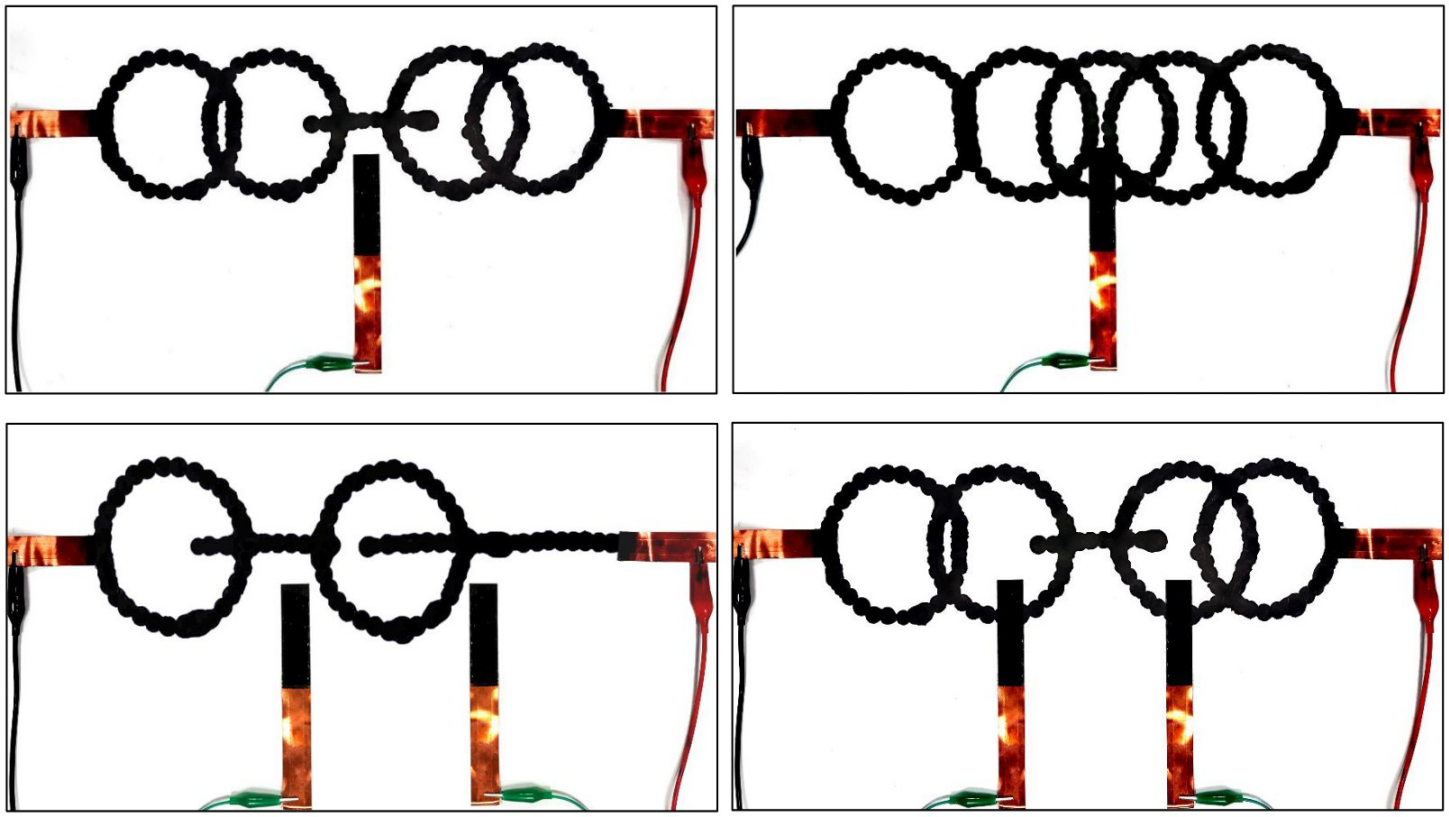
Examples of circuit patterns drawn during optimization. The left patterns avoid obstacles and achieve high load voltage. The right ones touch the obstacles and thus show a low voltage although they have more circles.

We further challenge CircuitBot with different obstacle patterns. Two obstacles were placed respectively at 130 *mm* and 250 *mm* according to the axis, in Fig 3. The results are shown in Fig 8. CoCaBO maintains superiority to BO and GA in respectively 21.3% and 25.7% higher in load voltage. Examples of circuit patterns that were drawn during the optimization process are shown in Fig 9.

## Discussion

### Superiority of CoCaBO over BO and GA

In our experiments GA showed the worst performance solving the circuit optimization problem. In a comparison between CoCaBO and BO, CoCaBO adopts a multi-armed bandit system to cope with categorical values, which directly gives the probability of each categorical choice. In contrast, due to the nature of Gaussian Processes embedded in BO, categorical variables have to be treated as continuous in order to generate probability distributions. From binary categorical variables, with two choices [0, 1] to a continuous space (0, 1), this conversion significantly increases the search space making it more difficult to find optimal solutions given limited trials. By combining Figs 6 and 8, we can see an obvious exploration stage in the parameter search space before iteration 18. The values of parameters change significantly from iteration to iteration, which provides the algorithm with information of different regions in the search space. With a more comprehensive knowledge of the problem, CoCaBO is more likely to find a global optimal. After the exploration stage, CoCaBO moves to an exploitation stage. Parameters are constrained in very small ranges where the algorithm finds the most promising to find solutions. In other words, as *s*_2_ and *s*_4_ are chosen to be circles, *x*_1_ and *x*_3_ (i.e. the center of the shape) are constrained in a very small area to allow *s*_2_ and *s*_4_ to steer away from the obstacle. Overall, CoCaBO manages the trade-off between exploration and exploitation well, which is the key to achieve high performance in budget limited experiments. Although we compared those three algorithms in scenarios which involved one and two obstacles, we did not use an “obstacle free” scenario as the system would not have any incentive to adopt straight lines in the solution.

### Importance of circuit drawing for future robots

To the best of our knowledge, this is the only work where a robot learns to optimize circuits with conductive ink to receive more energy. In previous approaches with robots charging themselves, works like [7, 8] demonstrated the ability of robots plugging themselves to recharge. However, robots can not always rely on outlets to recharge themselves (e.g. search and rescue missions and other unstructured environments). The circuits drawn with conductive ink have much higher flexibility and can be even three-dimensionally printed on walls and ceilings, further increasing the likelihood of robots connecting themselves to potential power sources and staying functional. In other works on harvesting energy with specially designed hardware [9, 12], the accumulation of energy is either slow or low in quantity. Meanwhile, the learning aspect of Robotics within their approaches was left aside. The crucial difference between those and the current work is that in our work the robot needs to learn to use tools to access power, while in the previous works this feature is already embedded to those robots.

Taking their own survival into consideration and adopting their lifespan as an objective function to be optimized, robots have an incentive to “live longer” and thus reach a higher level of autonomy. The stress of survival (e.g. limited energy, nearby hazardous areas, mechanical damages) could potentially trigger the learning of intelligent robot behaviors that are hard to program. It can be well foreseen that industrial robots self-optimize to achieve better performance in exchange for more energy; mobile robots and self-driving cars discourage themselves to attempt navigating through routes which are beyond their battery capacity; legged robots adjust themselves to new gaits after an injury; planetary rovers actively search energy to stay alive and avoid hazardous areas on the planet millions of miles away from the earth.

### Study limitations

The robotic arm has a limited workspace at which the power supply is accessible at all times, and this condition may differ from a real-case where this robot needs to power itself. Mobile robots equipped with a drawing system, instead of a liquid ink, could be a better fit for real-cases, as our experiments required a 30 minutes delay for the ink to dry. Another limitation is that the robot is not directly powered by the circuit it is optimizing, and this is because the graphene-based conductive ink we used has a lower conductivity than metal wires (when our robot moves many joints at the same time the current requirement exceeds the maximum current). Considering current technological limitations the application of this robot on a real problem are very limited, but advances in material science toward graphene-based 3D printed wires and self-healing circuits will enable our methods to be deployed in inhospitable environments. This work is the first to successfully deploy and instantiate this system using current technological resources, and in the future our results should be used to pave a creative way for robots to access ambient energy and stay functional.

## Conclusion

This paper presents the first robot capable of accessing and optimizing its energy intake through self-drawn electrical connections. With properly designed optimization routines, the robot can receive maximum power while avoiding obstacles within a small number of trials. Although our experiments are performed in a simplified environment, using conductive ink (a mean) and a power source (an end), this cable-free approach enables flexible deployment in complex environments, which is a very useful proof of concept for autonomous robots (e.g. in search-and-rescue missions). As robots become more present in our society and even reach other planets, maximizing the capacity to stay alive is crucial to increase the odds of success.

Apart from energy, other resources are also crucial for robotic survival. Keeping the usage of material (e.g., conductive ink) in the optimization routine is a further direction to explore. For example, circles outperform lines in reducing resistance of the connection but cost more ink. The robot should consider the trade-off between the usage of resources and the improvement in received energy. Currently, we only implement circuit drawing on flat surfaces. Applying circuit drawing in 3-dimensional space would be another interesting further direction.

## Supporting information

S1 Movie Experiment description. (MP4)

S1 Data (ZIP)
